# High-frequency electrical stimulation (HFES) data lean and obese Zucker rat tibialis anterior muscle: Regulation of glycogen synthase kinase 3 beta (GSK3B) associated proteins

**DOI:** 10.1016/j.dib.2017.11.036

**Published:** 2017-11-13

**Authors:** Gautam K. Ginjupalli, Kevin M. Rice, Anjaiah Katta, Nandini D.P.K. Manne, Ravikumar Arvapalli, Miaozong Wu, Shinichi Asano, Eric R. Blough

**Affiliations:** a Center for Diagnostic Nanosystems, Marshall University, Huntington, WV, USA; b Department of Internal Medicine, Joan C. Edwards School of Medicine, Marshall University, Huntington, WV, USA; c Biotechnology Graduate Program West Virginia State University, Institute, WV, USA; d Department of Health and Human Service, School of Kinesiology, Marshall University, Huntington, WV, USA; e Department of Public Heath, Marshall University, Huntington, WV, USA; f College of Health, Science, and Technology, University of Central Missouri, Warrensburg, MO, USA; g School of Education, Health, and Human Performance, Fairmont State University, Fairmont, WV, USA; h Department of Pharmaceutical Sciences and Research, School of Pharmacy, Marshall University, Huntington, WV, USA; i Department of Pharmacology, Physiology and Toxicology, Joan C. Edwards School of Medicine, Marshall University, Huntington, WV, USA

**Keywords:** Diabetes, Skeletal muscle, High-frequency electrical stimulation (HFES), Zucker rat, Tibialis anterior, GSK3b

## Abstract

Anaerobic exercise has been advocated as a prescribed treatment for the management of diabetes: however, alterations in exercise-induced signaling remain largely unexplored in the diabetic muscle. Here, we compare the basal and the in situ contraction-induced phosphorylation of the AMPK, GSK3beta, and Shp2 in the lean and obese (fa/fa) Zucker rat tibialis anterior (TA) muscle following a single bout of contractile stimuli. This article represents data associated with prior publications from our lab (Katta et al., 2009; Katta et al., 2009; Tullgren et al., 1991) [1–3] and concurrent Data in Brief articles (Ginjupalli et al., 2017; Rice et al., 2017; Rice et al., 2017; Rice et al., 2017) [4–7].

**Specifications Table**

**Table:** 

Subject area	*Biology*
More specific subject area	*Diabetic skeletal muscle response to exercise*
Type of data	*graph, figure*
How data was acquired	*immunoblotting*
Data format	*analyzed*
Experimental factors	*A high-frequency electrical stimulation (HFES) was used to produce 10 sets of 6 contractions over a 22-minute period. Tissues were collected and protein was then isolated from tissue for western blot analysis.*
Experimental features	*TA obtained from Lean and Obese male Zucker rats were used in this experiment*
Data source location	*Huntington, WV USA*
Data accessibility	*Data is with this article and is related to articles published and in review*[1], [2], [3], [4], [5], [6], [7].

**Value of the data**

• The data presented in this Brief is vital to understanding the effect of diabetes on skeletal muscle mechanotransduction.
• This data gives insight into the how diabetes alters tissue response to stimuli.
• This data provides a more thorough understanding of the mTor pathway involvement in exercise mediated signaling in both diabetic and non-diabetic muscle tissue.

## 1 Data

### 1.1 AMPK

**Fig. 1:**
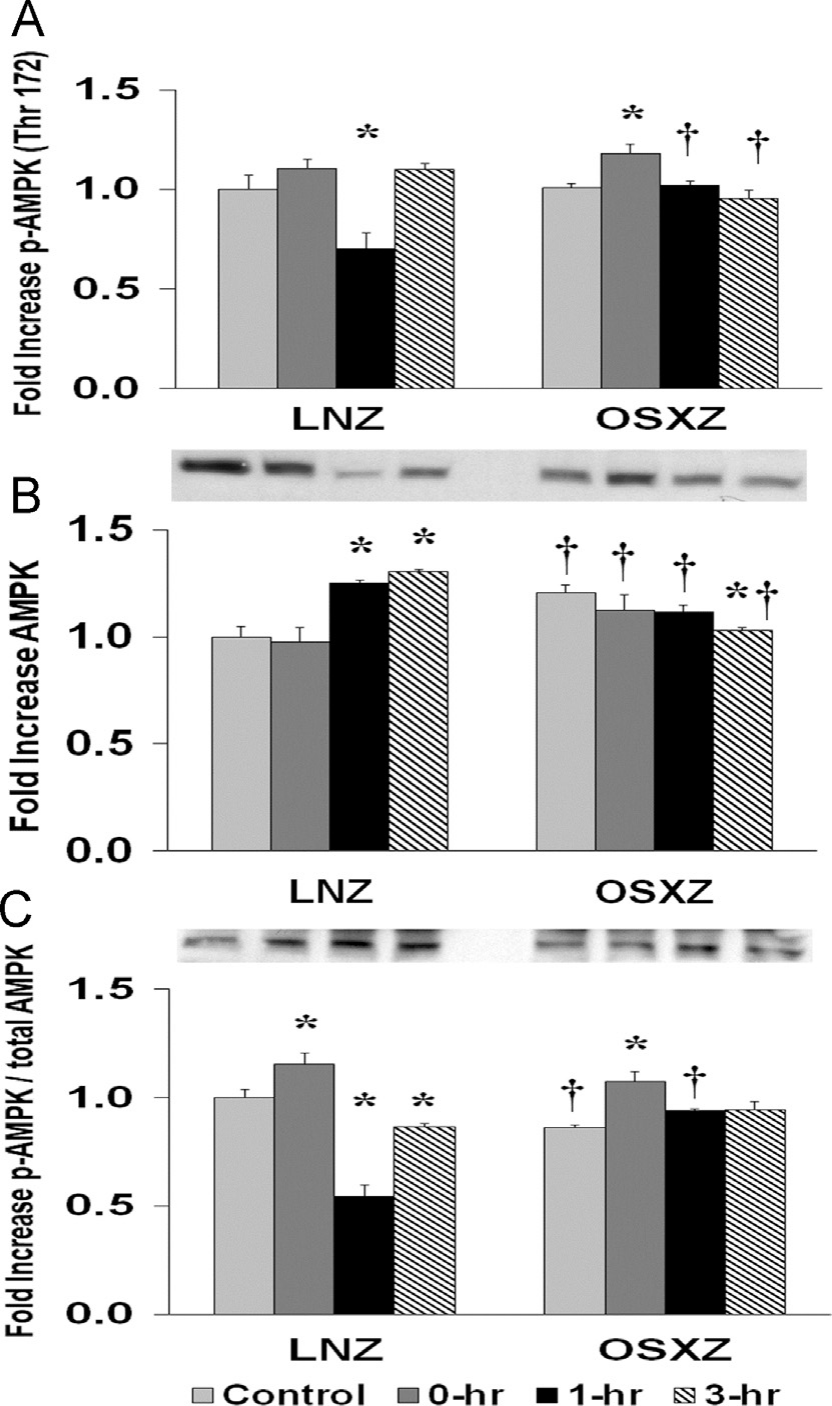
Diabetes alters HFES-induced expression and phosphorylation of AMPK rat TA. The basal (control) and HFES-induced expression of AMPK in TA from non-diabetic lean Zucker (LNZ) and diabetic obese syndrome X Zucker (OSXZ) rats. * Significantly different from HFES TA within the same group (*p*<0.05). † Significantly different from corresponding LNZ TA (*p*<0.05). *n*=6/group.

To determine the effect of HFES on soleus from diabetic male obese syndrome-X Zucker (OSXZ) diabetic and nondiabetic male normal lean Zucker (LNZ) animals we evaluated the expression of AMPK. TA basal AMPK content was higher (20.7±3.69%, *p*<0.05) in the OSXZ when compared to LNZ. HFES resulted in an increase in AMPK in the LNZ TA (24.9±1.4%, 30.3±1.3%, at 1 and 3 h, *p*<0.05) when compared to LNZ contralateral control. However, HFES resulted in an increase (17.7±1.3%, at 3 h, *p*<0.05) in the OSZX TA when compared to contralateral OXSZ control (Fig. 1).

To determine the effect of HFES on TA from OSXZ and LNZ animals we evaluated the phosphorylation of AMPK at Threonine 172. TA basal phosphorylation of AMPK Thr 172 demonstrated no difference in the OSXZ when compared to LNZ. HFES resulted in a decrease (29.8±7.9%, at 1 h, *p*<0.05) in phosphorylation of AMPK Thr 172 in the LNZ TA when compared to LNZ contralateral control. HFES resulted in an increase in phosphorylation of AMPK Thr 172 in the OSXZ TA (16.9±4.9%, at 0 h, *p*<0.05) when compared to OSXZ contralateral control (Fig. 1).

To determine the effect of HFES on TA from OSXZ and LNZ animals we evaluated the phosphorylation of AMPK Thr 172 to total AMPK. TA basal phosphorylation of AMPK Thr 172 to total AMPK was lower (13.7±1.1%, *p*<0.05) in the OSXZ when compared to LNZ. HFES resulted in an increase (15.4±5.3%, at 0 h, *p*<0.05) and a decrease (45.5±5.0% and 13.5±1.5%, at 1 and 3 h, *p*<0.05) in phosphorylation of AMPK Thr 172 to total AMPK in the LNZ TA when compared to LNZ contralateral control. HFES resulted in an increase in phosphorylation of AMPK Thr 172 to total AMPK in the OSXZ TA (21.1±4.5%, at 0 h, *p*<0.05) when compared to OSXZ contralateral control (Fig. 1).

### 1.2 GS3K-β

**Fig. 2:**
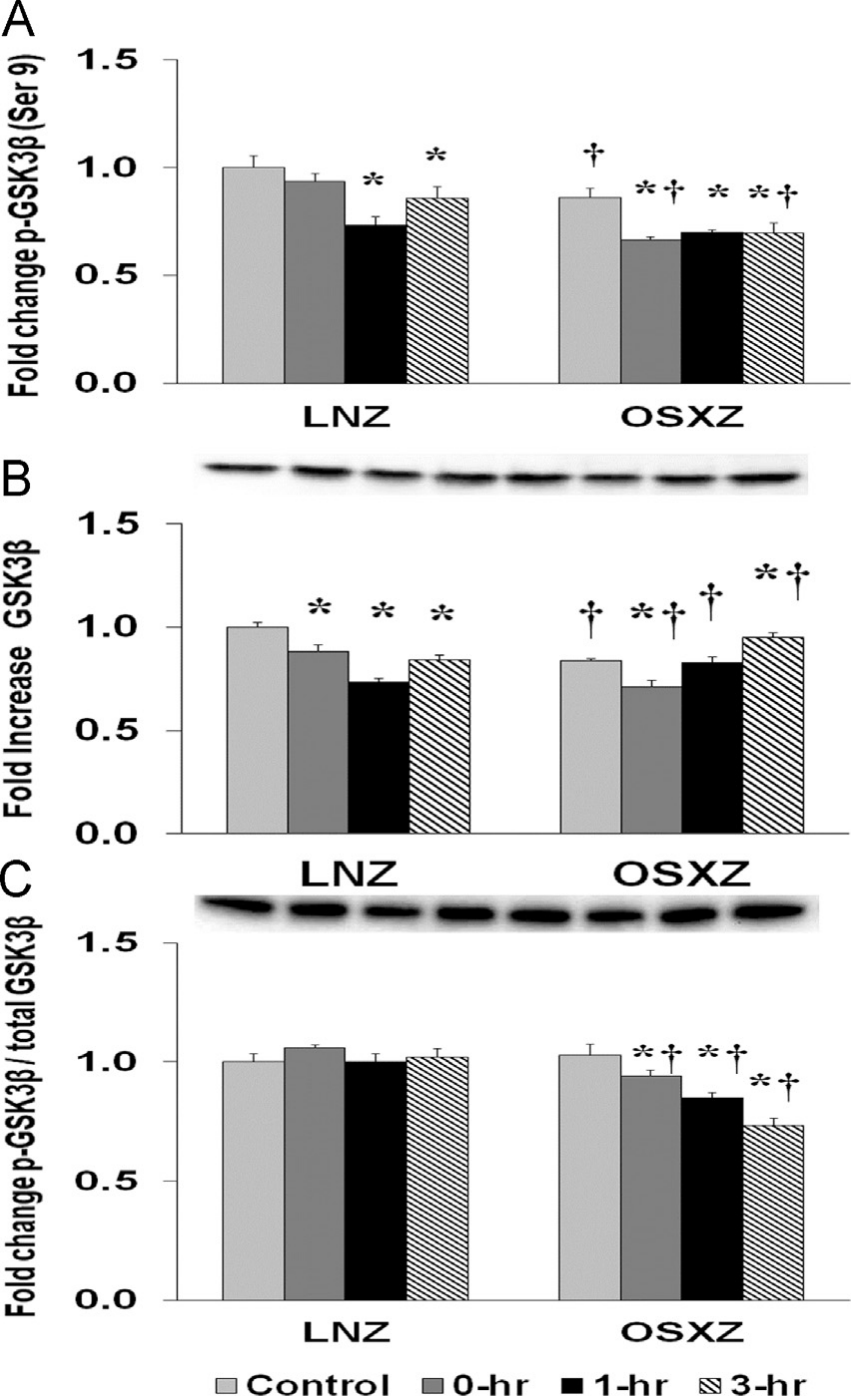
Diabetes alters l HFES-induced expression and phosphorylation of GSK3β rat TA. The basal (control) and HFES-induced expression of GSK3β in TA from non-diabetic lean Zucker (LNZ) and diabetic obese syndrome X Zucker (OSXZ) rats. * Significantly different from HFES TA within the same group (*p*<0.05). † Significantly different from corresponding LNZ TA (*p*<0.05). *n*=6/group.

To determine the effect of HFES on TA from OSXZ and LNZ animals we evaluated the expression of GS3K-β. TA basal GS3K-β content demonstrated a decrease (16.2±0.5%, *p*<0.05) in the OSXZ when compared to LNZ. HFES resulted in a decrease (11.6±2.9%, 26.8±1.9%, and 15.9±2.3%, at 0, 1 and 3 h, *p*<0.05) in GS3K-β in the LNZ TA when compared to LNZ contralateral control. HFES resulted in a decrease (12.8±3.0%, at 0 h, *p*<0.05) and an increase (11.0±2.6%, at 3 h, *p*<0.05) in the OSZX TA when compared to contralateral OXSZ control (Fig. 2).

To determine the effect of HFES on TA from OSXZ and LNZ animals we evaluated the phosphorylation of GS3K-β at Serine 9. TA basal phosphorylation of GS3K-β Ser 9 was lower (14.0±4.4%, *p*<0.05) in the OSXZ when compared to LNZ. HFES resulted in a decrease (26.7±3.7%, and 14.3±5.3%, at 1 and 3 h, p<0.05) in phosphorylation of GS3K-β Ser 9 in the LNZ TA when compared to LNZ contralateral control. HFES demonstrated a decrease (19.5±1.3%, 16.2±1.3%, and 16.3±4.6%, at 0, 1 and 3 h, *p*<0.05) in GS3K-β Ser 9 in OSXZ TA when compared to OSXZ contralateral control (Fig. 2).

To determine the effect of HFES on TA from OSXZ and LNZ animals we evaluated the phosphorylation of GS3K-β Ser 9 to total GS3K-β. TA basal phosphorylation of GS3K-β Ser 9 to total GS3K-β was not significantly different in the OSXZ when compared to LNZ. HFES elicited no significant change in phosphorylation of GS3K-β Ser 9 to total GS3K-β in the LNZ TA when compared to LNZ contralateral control. HFES demonstrated a decrease (8.6±2.6%, 17.7±2.1%, and 29.4±2.9%, at 0, 1 and 3 h, *p*<0.05) in phosphorylation of GS3K-β Ser 9 to total GS3K-β in the OSXZ TA when compared to OSXZ contralateral control (Fig. 2).

### 1.3 SHP2

**Fig. 3:**
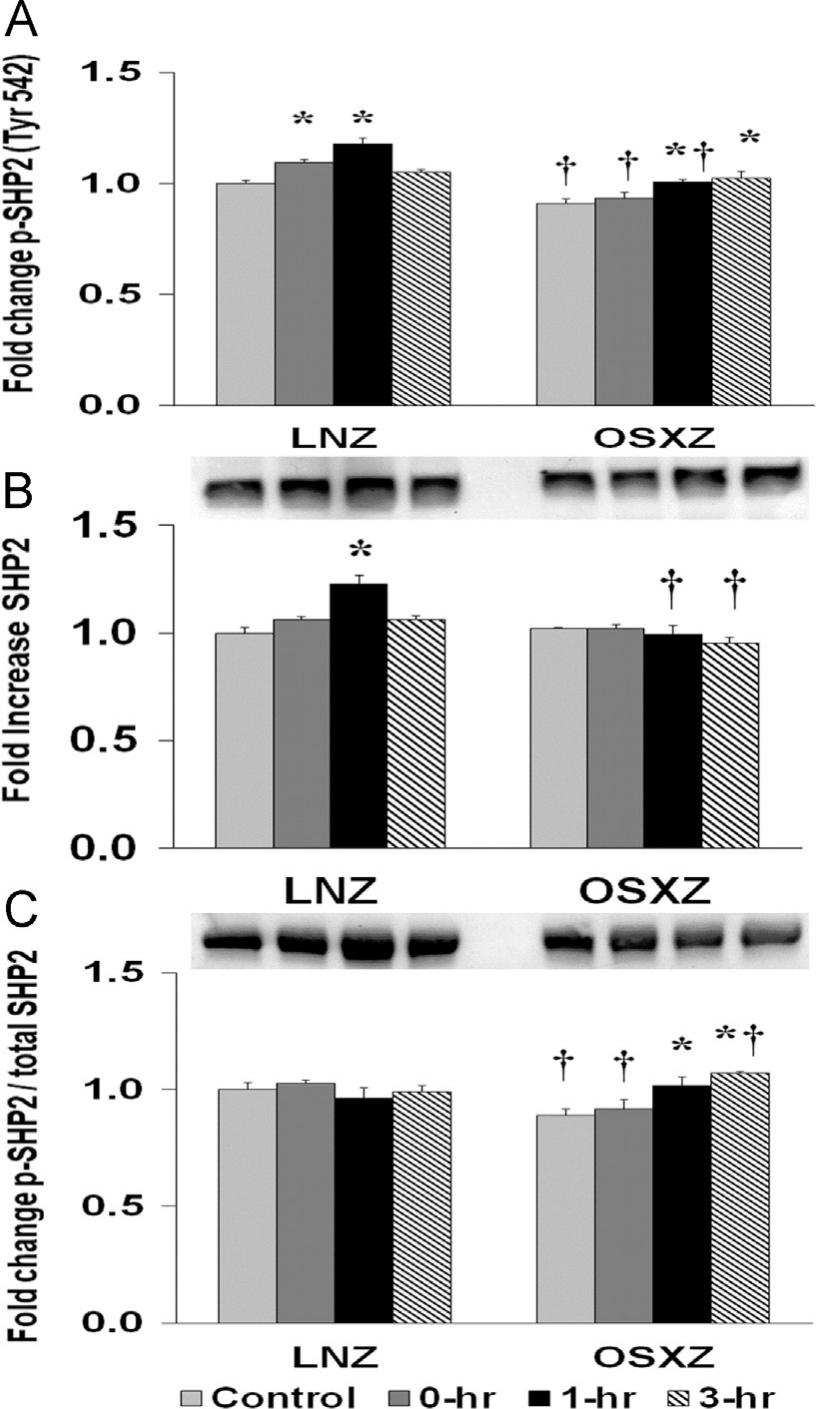
Diabetes alters l HFES-induced expression and phosphorylation of SHP2 rat TA. The basal (control) and HFES-induced expression of SHP2 in TA from non-diabetic lean Zucker (LNZ) and diabetic obese syndrome X Zucker (OSXZ) rats. * Significantly different from HFES TA within the same group (*p*<0.05). † Significantly different from corresponding LNZ TA (*p*<0.05). *n*=6/group.

To determine the effect of HFES on TA from OSXZ and LNZ animals we evaluated the expression of SHP2. TA basal SHP2 content was not significantly different in the OSXZ when compared to LNZ. HFES resulted in an increase (22.8±3.9%, at 1 h, *p*<0.05) in SHP2 in the LNZ TA when compared to LNZ contralateral control. HFES did not elicit a change in SHP2 in the OSZX TA when compared to contralateral OXSZ control (Fig. 3).

To determine the effect of HFES on TA from OSXZ and LNZ animals we evaluated the phosphorylation of SHP2 at tyrosine 542. TA basal phosphorylation of SHP2 Tyr 542 was lower (9.1±2.1%, *p*<0.05) in the OSXZ when compared to LNZ. HFES resulted in an increase (9.4±1.3% and 17.9±2.5%, at 1 and 3 h, *p*<0.05) in phosphorylation of SHP2 Tyr 542 in the LNZ TA when compared to LNZ contralateral control. HFES resulted in an increase in phosphorylation of SHP2 Tyr 542 in the OSXZ TA (9.8±0.9 and 11.6±2.9%, at 1 and 3 h, *p*<0.05) when compared to OSXZ contralateral control (Fig. 3).

To determine the effect of HFES on TA from OSXZ and LNZ animals we evaluated the phosphorylation of SHP2 Tyr 542 to total SHP2. TA basal phosphorylation of SHP2 Tyr 542 to total SHP2 was lower (11.0±2.5%, *p*<0.05) in the OSXZ when compared to LNZ. HFES elicited no significant change in phosphorylation of SHP2 Tyr 542 to total SHP2 in the LNZ TA when compared to LNZ contralateral control. HFES resulted in an increase in phosphorylation of SHP2 Tyr 542 to total SHP2 in the OSXZ TA (12.6±3.9% and 18.1±0.4%, at 1 and 3 h, *p*<0.05) when compared to OSXZ contralateral control (Fig. 3).

## 2 Experimental design, materials and methods

### 2.1 Animals

All procedures were conducted in strict accordance with the Guide for the Care and Use of Laboratory Animals as approved by the Council of the American Physiological Society and the Animal Use Review Board of Marshall University. Young (10 week, *n*=12) male lean Zucker (non-diabetic) (LNZ) and young (10 week, *n*=12) male obese syndrome-X Zucker (diabetic) (OSXZ) rats were obtained from the Charles River Laboratories and barrier housed one per cage in an AAALAC approved vivarium. Housing conditions consisted of a 12H:12H dark-light cycle and the temperature was maintained at 22±2 °C. Animals were provided food and water *ad libitum*. Rats were allowed to recover from shipment for at least two weeks before the commencement of experimentation during which time the animals were carefully observed and weighed weekly.

### 2.2 Materials

AMPK (#2532), p-AMPK (Thr 172) (#2535), glycogen synthase kinase-3β (GSK-3β) (#9332), Ser 9 phosphorylated GSK-3β (#9336), SHP-2 (#3752), p-SHP-2 (Tyr 542) (cat #3751), Mouse IgG, and Rabbit IgG antibodies were purchased from Cell Signaling Technology (Beverly, MA). Enhanced chemiluminescence (ECL) western blotting detection reagent was from Amersham Biosciences (Piscataway, NJ). Precast 10% and 15% SDS-PAGE gels were purchased from Lonza (Rockland, ME). Enhanced chemiluminescence (ECL) western blotting detection reagent was purchased from Amersham Biosciences (Piscataway, NJ). Restore western blot stripping buffer was obtained from Thermo scientific (Rockford, IL) and 3T3 cell lysates from Santa Cruz Biotechnology (Santa Cruz, CA). All other chemicals were from Sigma (St. Louis, MO).

### 2.3 Contractile stimulation of skeletal muscles

The high-frequency electrical stimulation (HFES) model has been previously described [8] and was chosen on the basis of its efficacy in stimulating protein translation and muscle hypertrophy in vivo [9]. The HFES model used in the present study produced 10 sets of 6 contractions with an overall protocol time of 22 min. Animals were killed by a lethal dose of pentobarbital sodium at baseline, immediately following, 1 h or 3 h (*n*=6 normal, *n*=6 diabetic for 0, 1, and 3 h) after HFES. Once excised, muscles were blotted dry, trimmed of visible fat and tendon projections, weighed, immediately frozen in liquid nitrogen, and stored at −80 °C.

### 2.4 Immunoblot analysis

Skeletal muscles were snap-frozen in liquid nitrogen at the end of each experiment. Protein isolates were prepared from the collected venae cavae by pulverizing the samples under liquid nitrogen using a mortar and pestle and washed Three times with ice cold phosphate buffered saline (PBS). Immunoblotting performed as described by Rice et al. [1], [2], [3], [4], [5], [6], [7].

### 2.5 Data analysis

Data were analyzed using Sigma Stat 3.0 statistical software and the results are presented as mean±SEM. Two-way ANOVA followed by the Student-Newman-Keuls post-hoc testing to determine differences between groups. The level of significance accepted *a priori* was <0.05.
