# Supplementary material for: High-frequency electrical stimulation (HFES) data lean and obese Zucker rat tibialis anterior muscle: Regulation of glycogen synthase kinase 3 beta (GSK3B) associated proteins
Source: Data Brief. 2017 Nov 13;16:423–9. doi: 10.1016/j.dib.2017.11.036 (PMC5723350; doi:10.1016/j.dib.2017.11.036)
Supplement: Supplementary file 1 — Supplementary material [file mmc1.zip › Conflict of interests.pdf]

Conflict of interests

The authors declare that they have no competing interests.
